# Plasma Membrane Receptors Involved in the Binding and Response of Osteoclasts to Noncellular Components of the Bone

**DOI:** 10.3390/ijms221810097

**Published:** 2021-09-18

**Authors:** Divakar S. Karanth, Macey L. Martin, Lexie S. Holliday

**Affiliations:** 1 Department of Orthodontics, College of Dentistry, University of Florida, Gainesville, FL 32610, USA; dkaranth@dental.ufl.edu (D.S.K.); maceymartin@ufl.edu (M.L.M.); 2 Department of Anatomy & Cell Biology, College of Medicine, University of Florida, Gainesville, FL 32610, USA

**Keywords:** integrins, vacuolar H^+^-ATPase, V-ATPase, LRP1, bone remodeling, synaptotagmin, CD44, osteopontin, extracellular vesicles

## Abstract

Osteoclasts differentiate from hematopoietic cells and resorb the bone in response to various signals, some of which are received directly from noncellular elements of the bone. In vitro, adherence to the bone triggers the reduction of cell–cell fusion events between osteoclasts and the activation of osteoclasts to form unusual dynamic cytoskeletal and membrane structures that are required for degrading the bone. Integrins on the surface of osteoclasts are known to receive regulatory signals from the bone matrix. Regulation of the availability of these signals is accomplished by enzymatic alterations of the bone matrix by protease activity and phosphorylation/dephosphorylation events. Other membrane receptors are present in osteoclasts and may interact with as yet unidentified signals in the bone. Bone mineral has been shown to have regulatory effects on osteoclasts, and osteoclast activity is also directly modulated by mechanical stress. As understanding of how osteoclasts and other bone cells interact with the bone has emerged, increasingly sophisticated efforts have been made to create bone biomimetics that reproduce both the structural properties of the bone and the bone’s ability to regulate osteoclasts and other bone cells. A more complete understanding of the interactions between osteoclasts and the bone may lead to new strategies for the treatment of bone diseases and the production of bone biomimetics to repair defects.

## 1. Introduction

Bones are a stable foundation of the vertebrate body [1,2]. Barring injury or pathologies, each bone maintains shape and structural properties throughout adulthood, despite being constantly remodeled. Injury, in the form of an appendicular fracture, provides a dramatic example of the dynamism of the bone [3]. In a few weeks, as long as the ends of the bone are contained in the body and approximated, the bone will heal and even be stronger, although without proper setting and casting, the form and function may be reduced. In a pathological example, unchecked rheumatoid arthritis can trigger abnormal bone resorption and remodeling, leading to debilitating skeletal deformities [4]. In another serious pathology, certain types of cancer cells can take advantage of the normal bone dynamics by triggering abnormal bone resorption or formation, resulting in a favorable niche for cancer cells [5]. A common and serious disease arising from imbalance of bone resorption and bone formation is osteoporosis, which afflicts both women and men as they age, although women are more vulnerable. The bone is weakened due to excessive resorption compared with formation, and much morbidity and mortality result from fractures that are a consequence of a structurally compromised bone [6].

Cancellous bone turns over more slowly than trabecular bone [2]. Some regions of the skeleton are also turned over more rapidly, for example, the maxilla [7]. Others, such as the long bones of the arms and legs, are remodeled at a slower rate [7]. In all cases, during normal bone remodeling, the removal and replacement are performed in such a way as to maintain the strength of the existing bones, while maintaining their overall form and interactions with the joints, muscles, and other bones.

In addition, to provide structural support for vertebrates, the bone also serves as a major supply of calcium. In response to needs for circulating calcium and phosphate, resorption and formation are stimulated by regulation by the parathyroid hormone/1.25-hydroxyvitamin D_3_ signaling axis. The levels of these hormones fluctuate in response to soluble calcium and phosphate [8]. In women, estrogen also plays a role in regulating bone resorption [9]. Hormonal changes at menopause result in perimenopausal bone loss, which makes women more susceptible to osteoporotic fractures [6].

Bone remodeling is controlled by a complex web of regulators, including hormones, chemokines, and cytokines, all converging on the central regulatory molecules in bone biology—the membrane proteins, receptor activator of nuclear factor kappa B (RANK) [10,11], RANK ligand (RANKL) [12], and osteoprotegerin, a soluble protein that binds RANKL and prevents its interaction with RANK. RANK is expressed in osteoclasts and their precursors. Stimulation of RANK by RANKL, usually derived from osteocytes [13], stimulates regulatory pathways that result in osteoclastogenesis, osteoclast survival, and osteoclast bone resorptive activity [12,14,15,16]. As will be discussed in more detail below, recent data suggest that bone remodeling can be coupled through these molecules. Specifically, RANK in extracellular vesicles (EVs) shed by osteoclasts stimulates a RANKL reverse signaling pathway in osteoblasts, promoting bone formation [17].

Although the RANKL/RANK/osteoprotegerin signaling network is central to bone remodeling, the activity of bone cells is also controlled by their physical environment [1]. This includes signals that are present in the organic elements of the bone, in the mineral component of the matrix, and in response to mechanical forces exerted on them in their bone microenvironment [1]. Together, these signals act in concert with various intercellular signals and hormones to trigger necessary bone resorption and to produce coupling factors that stimulate bone formation to replace the bone that is removed by osteoclastic resorption [18]. Although much of the regulation of osteoclasts is indirect through osteocytes [19,20,21], osteoblasts [19,22,23], lining cells [19] and even immune cells [15], in this review article, we will focus on what is known about the direct interaction of osteoclasts with the bone, and outstanding questions in the area.

## 2. Osteoclasts: Specialized Bone-Resorbing Cells

**Figure 1:**
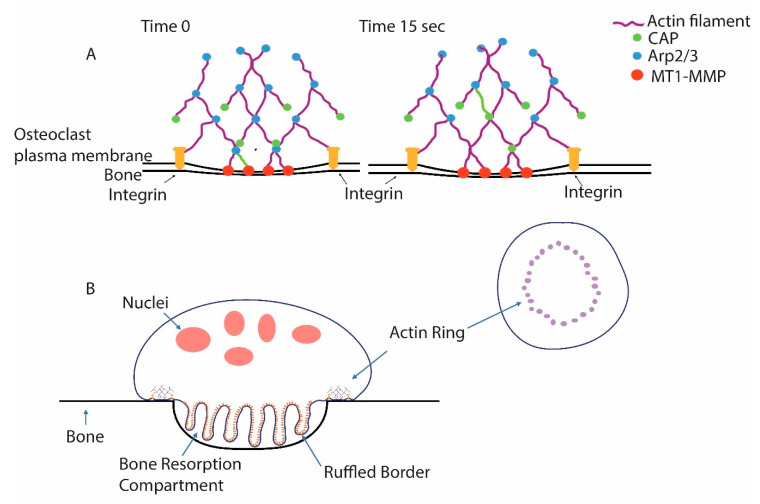
Podosomes are the core unit of the actin ring of osteoclasts. (**A**) Podosomes are discrete and dynamic microfilament-based structures that are associated with the ability of cells to invade the extracellular matrix. Constant actin polymerization is triggered by the actin-related 2/3 (Arp2/3) complex, which binds pre-existing filaments and triggers new filament formation. Capping protein (CAP) quickly binds the growing actin filaments’ end and prevents further growth, limiting the size of the individual filaments. Note that one actin filament is green and that, from time 0 to 15 s, it has finished growing and been capped and been translocated in the filament network from the plasma membrane where it polymerized. Podosomes are connected with the less dense actin filament network of the cell, which gives them purchase as the filament polymerization exerts force on the membrane. Integrins bind the matrix and hold the cells tight against the podosomes pushing against the membrane. Membrane type 1-matrix metalloproteinase (MT1-MMP) associated with the membrane degrades matrix proteins. (**B**) In osteoclasts, many podosomes are woven into a higher-order structure, the actin ring. Force from the podosomes of the actin ring pushes the membrane into the bone, forming a tightly sealed extracellular resorption compartment. Vacuolar H^+^-ATPases (V-ATPases) are inserted, as vesicles fuse with the nascent ruffled plasma membrane, and then pump protons into the resorption compartment, lowering the pH, solubilizing bone mineral, and providing an environment suitable for the protease activity of cathepsin K, which is secreted into the resorption compartment and is active in acidic environments. Cathepsin K is the primary agent for the degradation of the organic matrix of the bone.

Osteoclasts are cells that differentiate from hematopoietic cells that are closely related to dendritic cells [24]. They are highly specialized to invade a mineralized matrix [25]. Many cells have the capacity to degrade and migrate through the matrix, which involves cytoskeletal reorganizations to form dynamic structures called podosomes (also called invadopodia), the secretion of proteases, and sometimes the acidification of the matrix [26,27,28,29]. Degradation of the bone is a very specialized version of matrix degradation. To accomplish this, basic elements involved in matrix invasion found in other cell types are further organized into higher-order structures (Figure 1). Podosomes that are very similar or identical to those found in cell types like dendritic cells, or metastatic cancer cells, are woven together to form a structure called the actin ring [26,30,31]. The actin ring presses the membrane into the bone, forcing it to conform with the bone and thereby segregating an extracellular resorption compartment [30,32]. To oppose the pushing of the membrane into the bone, osteoclasts adhere to the bone using integrins (and perhaps other matrix receptors) that bind specific ligands in the organic matrix of the bone [33,34,35].

The plasma membrane segregated by the actin ring, called the ruffled border, is also highly specialized [26,32,36,37]. It is the site of the secretion of cathepsin K, the acid cysteine proteinase primarily responsible for degrading the organic matrix of the bone [38,39]. The ruffled border is packed with vacuolar H^+^-ATPases (V-ATPases), which pump protons from inside of the cell into the resorption compartment [40,41]. The ruffled border also contains chloride voltage-gated channel 7 (CLC-7) and its subunit, osteopetrosis-associated transmembrane protein 1 (Ostm1), which are required to dissipate the charge on the membrane that results from V-ATPases’ pumping of protons across the plasma membrane, and by doing so, allows sufficiently low pH values (<5) to be achieved for bone resorption to occur [42,43]. Mutations in the a3 subunit of V-ATPase, which is selectively expressed in osteoclasts and found in the ruffle membrane [44], CLC-7 [42], or Ostm1 [45], result in autosomal malignant osteopetrosis because of dysfunctional osteoclasts that are unable to resorb the bone.

The packing of V-ATPases into a plasma membrane domain is very atypical compared with most cells [46]. It occurs in osteoclasts, in α-intercalated and proximal tubule cells of the kidney, and in a few other cell types [47]. In most cell types, V-ATPases, which are essential housekeeping enzymes composed of 16 subunits [48,49], are expressed at low levels and excluded from the plasma membrane [46].

**Figure 2:**
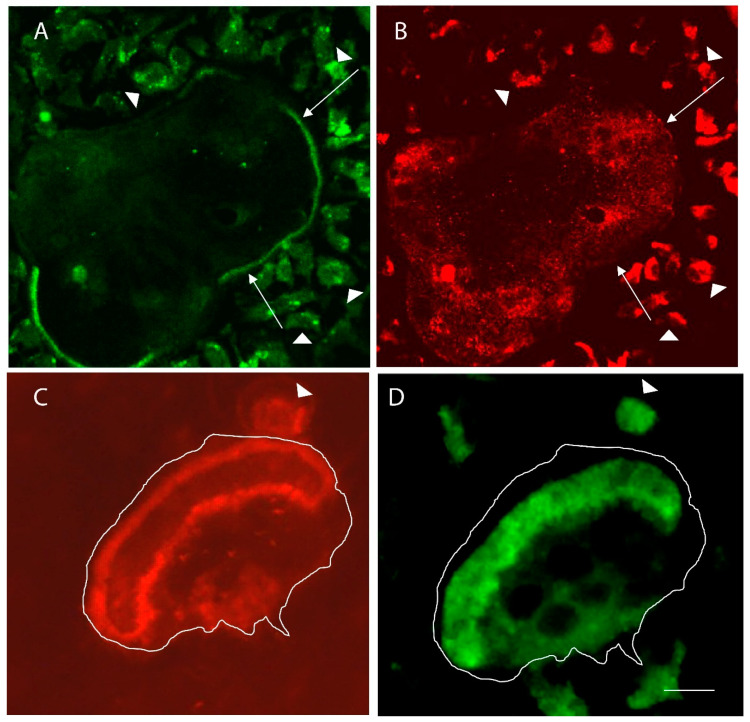
Comparison of inactive osteoclasts in glass substrate with resorbing osteoclasts in the bone. (**A**) Microfilaments, detected with phalloidin, of a large inactive osteoclast are integrated into podosomes that surround the periphery of the giant cell. Long arrows point to parts of the “actin belt” of podosomes. Arrowheads show small preosteoclasts in the area of the giant cell. (**B**) V-ATPase, detected with an anti-E-subunit antibody, shows the vesicular distribution in the giant cell, which is very flat (less than 2 microns thick). (**C**) The actin rings of two osteoclasts—the larger resorbing cell is outlined, and the arrowhead points to the actin ring of a smaller resorbing mononuclear osteoclast. (**D**) The V-ATPase is mostly packed into the ruffled borders of the resorbing cells, which are bounded by the actin rings. Scale bar equals 50 µm in (**A**,**B**) and 10 µm (**C**,**D**).

In cell culture, the fate of osteoclasts is dependent on the substrate. If osteoclasts are placed on plastic or glass, many of the osteoclasts continue fusing until they become “giant” cells, which often contain hundreds of nuclei. These cells have podosomes, but they are smaller and less dynamic than the podosomes found in the actin ring of resorbing osteoclasts [50,51], and they typically surround the periphery of the cells, forming an “actin belt” (Figure 2A). In addition, although these cells have high levels of V-ATPase, they do not insert large amounts into the plasma membrane; the V-ATPase is maintained in cytosolic storage vesicles (Figure 2B) [52]. In striking contrast, the same cells, when adhering to the bone or dentine, fuse less and instead form actin rings and ruffled borders and resorb the substrate (Figure 2C,D) [46,53]. The podosomes in the actin rings are larger and more dynamic, and podosomal microfilaments are interwoven with microfilaments to make the ring structure [31,50]. Interestingly, if osteoclasts on plastic are lifted and applied to the bone, within 24 h no giant cells (>20 nuclei) are detected, and instead, osteoclasts that rarely have more than 10 nuclei resorb the bone. One possibility is that the giant cells have undergone fission to form the smaller resorbing osteoclasts. This possibility is of particular interest because of a recent article identifying cells called “osteomorphs” that in vivo are derived from larger multinuclear osteoclasts by fission events, and which can be recycled by fusing to form larger osteoclasts [54]. These recently discovered cycles of fusion and fission were proposed to be a normal part of the life of osteoclasts in vivo. Their regulation is not understood, although the fusion events seem to require RANKL stimulation [54].

Cycles of activation followed by inactive periods have been reported [50,55]. One means by which activated osteoclasts can be rapidly inactivated is by treatment with inhibitors of phosphatidylinositol 3-kinase, such as wortmannin of LY294002 [53,56,57]. Inactivation involves disruption of the actin ring structure, which is coincidental with the retrieval of V-ATPase from the plasma membrane and binding between V-ATPase and microfilaments through an actin-binding domain in the B2 subunit of the V-ATPase [53,58]. This binding is almost certainly indicative of disassembly of V-ATPases into subdomains. The E-subunit and G-subunits form stator arms that cover the actin-binding sites in fully intact enzymes, making the interaction between the B2 subunit and microfilaments difficult to envision in the intact enzyme [48,49]. Moreover, it is well established that reversible assembly is a crucial regulatory mechanism controlling V-ATPase activity [59].

## 3. The Role of Integrins in Regulating Osteoclast Activity

What elements of the bone stimulate the activation of osteoclasts? One instructive set of experiments examined the role of metalloproteinases and acid cysteine proteinases in bone resorption in vitro, making use of calcitriol-stimulated mouse marrow, which contains both osteoclasts and osteoblasts, and which represents a model for the bone microenvironment. It was found that the inhibition of acid cysteine proteinases (which included cathepsin K) with E64 resulted in abnormal resorption pit formation [60]. The amount of bone surface area resorbed was the same as no proteinase inhibitor vehicle controls. However, although the pits formed were demineralized, the organic matrix was not removed. In contrast, when matrix metalloproteinases, and specifically interstitial collagenase, were inhibited, the surface area resorbed was dramatically reduced, although the pits that were formed were normal and had both the mineral and the organic matrix removed [60]. Activation of osteoclasts was restored to control levels in the presence of the matrix metalloproteinase inhibitor by precoating bone slices with either collagen that had been cleaved by collagenase or by heat-denatured type I collagen [60]. These data suggest that cleavage of type I collagen triggered the activation of osteoclasts. When type I collagen is cleaved, it is denatured, and this exposes arginine-glycine-aspartate (RGD) sequences that are bound by certain integrins. These RGD sequences are cryptic in the native collagen. This was proposed to be the activation signal triggered by interstitial collagenase activity. Subsequent studies have provided data that are consistent with this idea [61,62,63,64].

**Figure 3:**
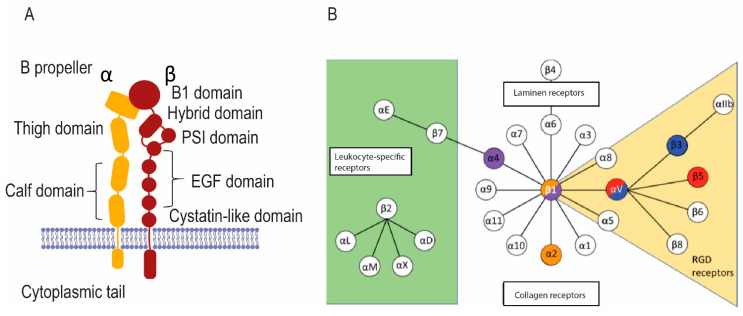
Integrins in osteoclasts bind to and detect signals in the bone matrix. (**A**) The general structure of integrins. Transmembrane α and β integrin proteins pair in various ways to make integrins with various specificities and signaling properties. (**B**) The chart shows various integrin pairs; colored integrins are found in osteoclasts.

Integrins, each of which is composed of a pair of membrane proteins designated α and β [35], are central adhesion molecules allowing cells to adhere to the extracellular matrix (Figure 3A). At the same time, integrins detect signals in the matrix and respond to those signals through various signal transduction pathways [65]. There are numerous α and β subunits that can associate with selected partners (Figure 3B) [66,67]. One important signaling molecule involved in integrin-based signaling is cellular src (c-src) [67]. This tyrosine kinase was identified as a proto-oncogene by a work, leading to a Nobel Prize for J. Michael Bishop and Harold Varmus [68]. C-src was found to accumulate at integrin adhesions and to be a crucial player in integrin-based signaling [69,70,71,72]. Because of its perceived importance, it was among the first genes to be knocked out when transgenic knockouts became possible. The knockout mice had relatively few pathologies, but they were osteopetrotic due to the failure of osteoclasts to resorb the bone properly [73,74,75].

Osteoclasts have integrins on their plasma membrane surface (Figure 3B). αVβ3 integrin, which binds RGD sequences in denatured collagen and other collagen-associated matrix proteins, including osteopontin and bone sialoprotein, is strongly upregulated during osteoclastogenesis [76,77]. The interaction between αVβ3 integrin and its ligands is implicated in the activation of osteoclasts [76,77,78,79,80,81]. Consistent with this, mice in which β3 integrin is knocked out are osteosclerotic, and their osteoclasts display aberrant morphology, unusual cytoskeletal organization, and reduced bone resorptive activity [82]. It is noteworthy, however, that osteoclasts lacking β3 integrin still adhere to and resorb the bone, although less effectively. The severity of the bone disorder is partially ameliorated by three times more osteoclasts than normal being present. The lack of β3 integrin reduces bone loss due to ovariectomy, a model for postmenopausal osteoporosis [83]. A specific mutation in β3 integrin that results in Glanzmann’s disease was also linked to osteopetrosis and impaired osteoclast function [84]. The primary pathology in Glanzmann’s disease is thrombasthenia, the result of the absence of β3 in platelets, resulting in their dysfunction [85].

αVβ5 integrins are also found in osteoclasts [86]. In contrast to β3 integrin, knockout of β5 integrin in mice results in increased osteoclast formation, increased bone resorptive activity, and decreased bone mineral density [87]. In addition, knockout of β5 integrin results in increased bone loss after ovariectomy. Taken together, these data suggest that αVβ5 integrin is a negative regulator of osteoclastogenesis and osteoclast resorptive activity [87]. It is of interest that the two αV-containing integrins in osteoclasts, αVβ3 and αVβ5, have opposite regulatory effects on osteoclasts, even though both are thought to bind the same RGD sequences.

Osteoclasts also have large amounts of α2β1 integrin, which binds native collagen [88,89]. This leads to the idea that as the bone surface is prepared by enzymes, such as interstitial collagenase, by altering native collagen to denatured collagen, osteoclasts change from receiving signals through the α2β1 pathway to αVβ3 and αVβ5, and this represents a component of the activation process [81]. This would represent a version of the concept of integrin switching [90,91].

## 4. Identification of a Minimal Substrate Required to Stimulate Osteoclasts to Produce Actin Rings and Ruffled Borders

Although responses by osteoclasts to matrix proteins coated onto plastic or glass can be observed, to our knowledge, there has not been a demonstration that active osteoclasts exhibiting both actin rings and V-ATPase-enriched ruffled borders occur in a nonmineralized matrix. Osteologic discs (BD Biosciences) are coated with a proprietary mineral layer, but no protein [92]. Even though osteoclasts, and also non-osteoclasts, have been shown to remove mineral from Osteologic discs [92], to our knowledge, the formation of a true ruffled border has not yet been demonstrated. Although widely used in the bone field, in our view these types of “resorption” assays must be viewed with some skepticism until documentation of V-ATPase-packed ruffled borders is provided.

RAW 264.7 osteoclast-like cells, formed by treating RAW 264.7 with recombinant RANKL, resorb Osteologic discs even though they do not (in our hands) form pits in the bone. Osteologic disc resorption has been shown by many groups. Bone resorption has been reported but is rarely convincingly verified by scanning electron microscopy. RAW 264.7 osteoclast-like cells in the bone form convincing actin rings, but have not been shown to make ruffled borders, although they insert V-ATPase into the plasma membrane [93].

We have sought to use the polymer-induced liquid precursor (PILP) process described by Gower and colleagues [94,95] to generate a minimal substrate required to stimulate both actin rings and ruffled borders [96]. This involves the use of proteins with long stretches of acidic acids, like those that occur in many noncollagenous matrix proteins associated with the bone [97]. Alternatively, a simple artificial protein consisting of a long string of polyaspartic acid can be employed. The acidic amino acids accumulate a shell of concentrated but noncrystalline Ca^2+^ molecules that can infiltrate a collagen matrix, which then allow calcium nanocrystals to form within the matrix [98].

When this process was used to mineralize thin slices of the demineralized bone matrix or densified collagen, the resulting bone biomimetic had calcium nanocrystals deposited in the matrix, giving it similar structural properties to slices of the bone or dentine, regardless of whether osteopontin (a noncollagenous matrix protein) or polyaspartic acid was used to drive to the PILP process [96]. However, the responses of osteoclasts to the biomimetics were strikingly different; although low levels of osteoclast activation (actin ring and ruffled border) occurred in the polyaspartic-acid-mineralized bone biomimetics, very high levels occurred in biomimetics mineralized with osteopontin [96]. This suggests that domains in osteopontin other than the polyaspartic-acid-rich domain were involved in stimulating the activation of osteoclasts in the context of the remineralized/demineralized bone matrix.

Osteopontin has RGD sequences and serves as a ligand for αVβ3 integrin, and this is a good candidate to be the activation signal [80]. Osteopontin is a complex protein that can be variably modified by O- and N-glycosylation, sulfation, phosphorylation, and transglutamination [99,100,101]. Osteopontin has a central region that contains both RGD- and non-RGD-binding sites for multiple integrins. Adjacent to the RGD motif is the sequence SLAYGLR (SVVYGLR in human), which serves as a cryptic binding site for α4β1, α4β7, and α9β1 integrins. It is masked in full-length osteopontin but is exposed following osteopontin cleavage by multiple proteases in tumors and sites of tissue injury [102,103]. Although osteoclasts have not been reported to have these integrins, we detected α4 integrin in extracellular vesicles shed by osteoclasts in the bone or dentine [104]. This suggests that either the osteoclasts or osteoclast precursors have α4β1 integrin and may detect the SLAYGLR sequence. However, knockout of α4 integrin does not lead to a dramatic bone phenotype [105,106].

In summary, data show that integrins play a role in the activation of osteoclasts to resorb the bone. A vital element is the interaction between αVβ3 integrin, which is upregulated during osteoclast formation, and RGD sequences in the bone matrix. However, the fact that knockout of β3 integrin does not completely block bone resorption [82] suggests that there are redundancies in the system. Data also suggest that osteoclasts receive negative signals from αVβ5 integrin and that switching from binding and stimulation through α2β1 integrin to αVβ3 may play a role in the activation process. Finally, data support the idea that integrin stimulation alone is not sufficient to account for the osteoclast activation response and may not even be required.

## 5. Other Membrane Receptors Involved in Regulating Osteoclast Activation

### 5.1. CD44 and IgSF11

CD44 is a glycosylated type I transmembrane protein that is ubiquitously expressed in higher mammals [107]. It was originally identified as a hyaluronic acid receptor and was subsequently shown to bind other extracellular matrix components, including collagen, laminin, fibronectin, and osteopontin. In various cell types, evidence has been presented that suggests that CD44 is involved in the regulation of cell adhesion, cell motility, matrix degradation, cell proliferation, and cell survival [107].

CD44 is found in osteoclasts, and studies have suggested that CD44 plays a crucial role in osteoclasts [108,109]. For example, in vitro it was shown to regulate osteoclast differentiation and fusion and to mediate the effects of osteopontin in osteoclasts [110]. However, transgenic knockouts of CD44 did not show a bone phenotype [111]. Knockout of CD44 enhanced cell fusion in plastic but not in the bone. More recently, CD44 was shown to have a compensatory effect on knockout of immunoglobulin superfamily 11 (IgSF11), a calcium-dependent cell–cell adhesion molecule [112]. IgSF11 knockout mice were recently shown to have high bone mass. The ability of CD44 to compensate for IgSF11 deficiency was apparently due to the interaction of both CD44 and IgSF11 with PSD-95, a scaffolding protein on the cytoplasmic face of cell adhesions [112]. These data suggest that, in osteoclasts, CD44, with its ability to interact with extracellular matrix ligands, might also interact with cell–cell adhesion molecules such as IgSF11 in the coordination of matrix binding and cell fusion. In the case of the osteoclast, where cell fusion (and fission) varies depending on the substrate, this may be a crucial regulatory activity [54]. If so, this regulation involves redundancies. Further studies will be required to examine and clarify these questions.

### 5.2. Low-Density-Lipoprotein-Related Protein 1 (LRP1, Also Known as CD91 and α-2-Macroglobulin Receptor)

Recently, LRP1 was identified as a membrane receptor involved in osteoclast formation and bone remodeling [113,114,115,116]. LRP1 is involved in receptor-mediated endocytosis [117]. It has been linked to lipoprotein metabolism, cell motility, and neurodegenerative diseases, atherosclerosis, and certain types of cancers [118]. Osteoclast-selective knockout of LRP1, using the cathepsin K promoter to drive a cre/lox system, resulted in mice with a low bone mass phenotype [115]. In contrast, in cell culture, knockdown of LRP1 in RAW 264.7 cells yielded reduced numbers of osteoclast-like cells and reduced the inhibition of osteoblasts by the conditioned media from the knockdown cells [113].

**Figure 4:**
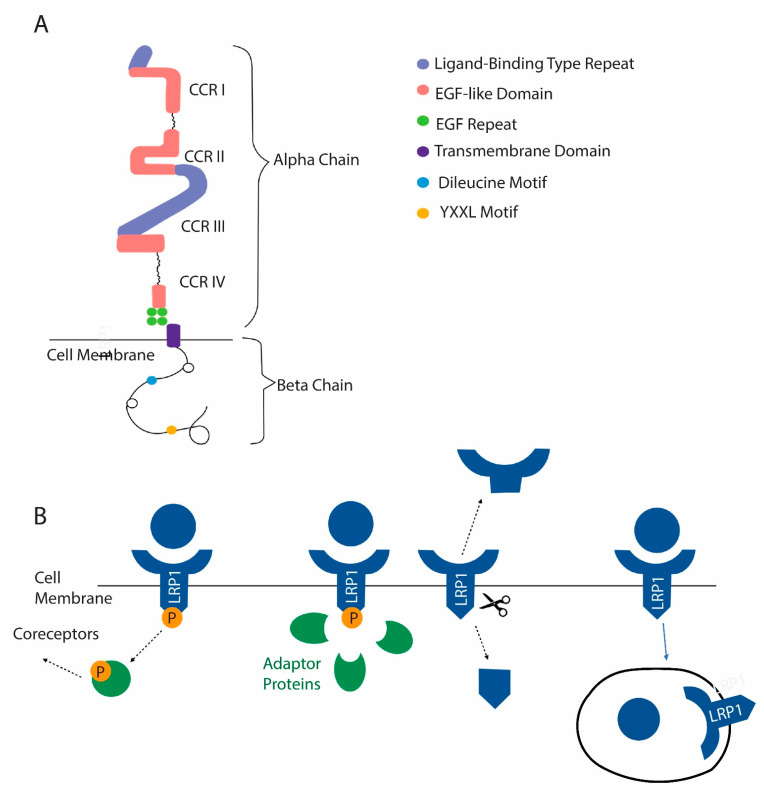
LRP1 is a newly identified membrane receptor with a role in osteoclast function. (**A**) Domain structure of LRP1. (**B**) Mechanisms by which LRP1 functions include the activation of coreceptors after ligand binding. The activation of signaling pathways after recruitment of adapter proteins can occur in response to ligand binding. LRP1 can be cleaved, releasing a soluble extracellular domain and a cytosolic domain that can act in the nucleus. Finally, LRP1 can act as a scavenger receptor. After certain ligands are bound, they are internalized with LRP1.

LRP1 is first synthesized as a 600 kD precursor, which is processed by furin to produce a 515 kD α chain and an 85 kD β chain (Figure 4A) [117]. The α chain is the extracellular domain, and the β chain is intracellular. In its extracellular domain, it has four distinct cysteine-rich complement-type repeats, each of which interacts with various extracellular ligands (Figure 4B). These include extracellular matrix proteins, including fibronectin- and heparin-sulfate-containing proteoglycans and various proteinases, including cysteine proteinases and matrix metalloproteinases. Both proteinases complexed with inhibitors, including α-1-antitrypsin, and uncomplexed proteinases bind LRP1. It also interacts with growth factors, including transforming growth factor β (TGF β) and bone morphogenetic factor 4 (BMP4), which are involved in bone modeling and remodeling. The β chain binds endocytic and scaffold adapters, including disabled-1, FE-65, and postsynaptic density protein [117]. These link LRP1 to membrane-bound proteins, including amyloid precursor protein, and are involved in various signal transduction pathways [118]. LRP1 can also undergo intramembranous proteolysis, which releases an extracellular LRP1 fragment and a cytosolic domain that is imported into the nucleus, where it directly regulates gene expression.

The size and complexity of LRP1 make it challenging to study. The current data suggest that it is an important and relatively unstudied means by which osteoclasts interact with the bone.

## 6. Matrix Receptors in Extracellular Vesicles (EVs) Released by Osteoclasts

EVs, vesicles with a diameter of 30–150 nm released by cells, have recently emerged as intercellular regulators involved in bone remodeling [119]. EVs containing RANKL have been shown to stimulate osteoclast formation [120,121]. EVs from osteoclasts that contain RANK have been shown to regulate bone cells in vitro [122] and to couple bone resorption with bone formation in vivo [17]. Studies also suggest that EVs from osteoclasts contain microRNA-214-3p, which serves to regulate osteoblasts [123,124].

**Figure 5:**
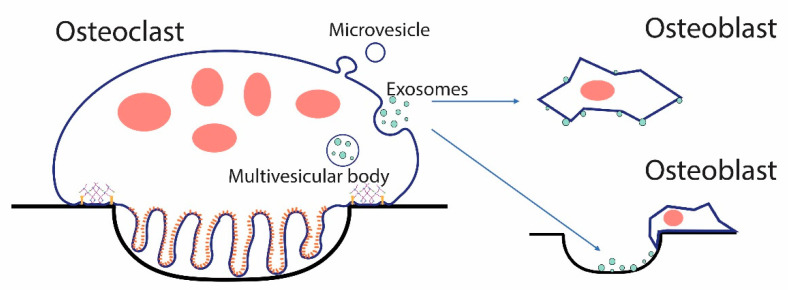
EVs from osteoclasts could signal osteoblasts and other cells directly, or could bind the bone or extracellular matrix serving as matrix-associated signaling units. Osteoclasts can shed two types of EVs, exosomes, which are released when the multivesicular body fuses with the plasma membrane. Microvesicles bud directly from the plasma membrane. In either case, EVs can directly interact with target cells or can bind the bone, as shown in a resorption pit above, and other forms of extracellular matrix through membrane receptors such as integrins, and the bound form can then interact with cells.

Various integrins are found in EVs shed by osteoclasts [22,104]. Integrins in EVs have been implicated in the organotropism of EVs [22,125,126,127]. Presumably, integrins in EVs interact with the matrix in specific organ niches such as the bone (Figure 5). Once bound to the matrix, the EVs could then interact with local cells, either through surface ligands such as RANK also present in the EVs or by fusion with cells and the delivery of membrane or luminal contents into the target cells. Integrins have also been shown to be involved in the migration of cancer cells by “decorating” the matrix with attachment sites [128,129,130].

Quantitative proteomic examination of EVs shed by osteoclasts showed that LRP1 was among the most abundant proteins found [104]. LRP1 was more abundant in EVs from osteoclasts resorbing the dentine than osteoclast resorbing the bone. This suggests that like RANKL [11], and the (pro)renin receptor [131], LRP1 represents another protein where its extracellular domain can be shed as a soluble protein, or the intact protein can be shed in EVs. This opens the door to a bewildering array of possibilities for regulatory effects. For example, LRP1 in the plasma membrane might either trigger endocytosis of a proteinase or stimulate cytosolic signaling pathways. In contrast, LRP1 in an EV might bind the same proteinase, block its internalization and clearance, and, through another receptor (for example, an integrin), bind it to the matrix where the proteinase is positioned to degrade matrix proteins.

CD44 is also found in EVs released by osteoclasts and, like RANK, is enriched in EVs from osteoclasts resorbing the bone [104]. Interest in CD44 in EVs has been stimulated by the finding that CD44-containing EVs serve as a biomarker for glioblastoma [132]. In addition, specific microRNAs have been detected in the glioblastoma-derived CD44-containing EVs, and evidence was presented suggesting that these may prove to be enhanced diagnostic markers for this devastating disease [132]. Since specific microRNAs from osteoclast EVs, particularly miR-214-3p [123,124], have been implicated in their regulatory function, it will be of great interest to determine whether the packaging of specific microRNAs into EVs is tied to the membrane receptor content of the EVs.

## 7. Calcium and Osteoclasts

As described above, there have been many efforts to determine the minimal requirements for triggering the activation of osteoclasts, which we define as having both an actin ring and a V-ATPase-packed ruffled border. In addition to examining osteoclasts of various nonproteinaceous calcium substrates, extensive efforts testing nonmineralized substrates have been performed. To our knowledge, none has proven to be able to stimulate ruffled borders, and thus to be true osteoclast activators. For example, we were not able to show convincing evidence that activation occurred in demineralized bone, while extensive activation of osteoclasts occurred in the same matrix after it had been mineralized, making use of osteopontin to drive the PILP process [96]. Whether activation requires a stiffness of the substrate, the calcium-containing crystals, or some combination is not clear.

One challenge is that there are relatively few quality antibodies available to detect ruffled border elements (V-ATPase subunits, CLC-7, OSTM1). Often studies are conducted without this crucial piece of information. However, based on our own studies and the literature, substrates such as type I collagen, osteopontin, and other matrix proteins coated onto glass or plastic do not stimulate osteoclasts to activate. It seems likely that either because of the specific texture and rigidity of the surface due to nanocrystals of hydroxyapatite and/or because of the calcium released that calcium is required.

**Figure 6:**
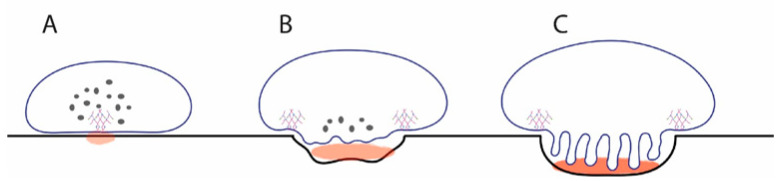
Model for how release of calcium at initial podosome patch could stimulate vesicle fusion to generate a ruffled border. (**A**) Initial patch of microfilaments and V-ATPase locally lowers pH sufficiently to release soluble calcium (light brown). Most V-ATPases are in cytosolic V-ATPase storage vesicles. (**B**) Calcium detected by synaptotagmin VII triggers fusion of cytosolic vesicles, introducing V-ATPases into the emerging ruffled border. This pumps more protons into the resorption compartment, leading to more release of soluble calcium. (**C**) Over time, this process continues until the mature resorption compartment of the resorbing osteoclast is formed, leaving no V-ATPase storage vesicles in the cytosol. The osteoclasts maintain low levels of other subsets of V-ATPase in the endosomal system for housekeeping functions.

Dynamic video studies of live osteoclasts, containing GFP-tagged actin, “resorbing” a hydroxyapatite substrate showed that actin rings began with a patch of podosomes, which gradually opened up into the ring structure and later closed like a purse string back to a patch, which then disappeared [50]. Although in that study a ruffled border was not demonstrated, similar cytoskeletal changes, determined by images at specific time points after osteoclasts were experimentally inactivated or reactivated using phosphatidylinositol-3-kinase inhibitors, occurred in resorbing osteoclasts in the bone, which tracked both microfilaments and V-ATPase [53]. In osteoclasts in the bone, podosomal patches accumulated at the same place and time as patches of V-ATPase, and likewise reuptake of V-ATPase from the plasma membrane involved first patching of both podosomes and V-ATPase [53]. The patching of F-actin and V-ATPase could further be explained by the direct binding interaction between microfilaments and the B2 subunit of V-ATPase [58,133]. It is plausible that at the initial patching stage, sufficient calcium may be released by the actions of the podosomes and V-ATPase acidification to drive the expansion of the actin ring and the formation of the ruffled border due to calcium-stimulated fusion of cytosolic vesicles with the developing ruffled plasma membrane (Figure 6). This model also suggests that a calcium sensor, which is linked to vesicle fusion, is present to stimulate this process. As described below, synaptotagmin VII is a strong candidate for this role [134].

Synaptotagmin VII is one of 15 isoforms of synaptotagmin [135,136]. It has been shown to regulate calcium-dependent exocytosis in fibroblasts and neurons, dense-core vesicles in PC12 cells, insulin secretory granules in pancreatic islet β-cells, secretory lysosomes in cytotoxic lymphocytes, and lysosome membrane fusion in macrophages [135]. In osteoclasts, knockout of synaptotagmin VII results in osteopenia and a low bone mineral density phenotype [134]. The underlying pathology of synaptotagmin VII knockout mice was low turnover bone remodeling with less active than normal osteoclasts.

Synaptotagmin VII localizes to vesicles in the cytosol of osteoclasts, and as the osteoclasts activates, it is concentrated in the ruffled border exactly like V-ATPase and is therefore properly localized to regulate ruffled border formation through the fusion of cytosolic vesicles with the plasma membrane [134]. Despite the potential importance of this finding, many questions remain. For example, is there a direct interaction between V-ATPase and/or CLC-7 and synaptotagmin VII? The packaging of these proteins into the ruffled border suggests that this may be the case. A previous study showed that V-ATPases in the ruffled border of resorbing osteoclasts displayed strong resistance to solubilization by Triton X-100 detergent extraction, remaining coherent for 10 min after extraction [52]. This hints that lateral associations between V-ATPases with each other or with other proteins in the ruffled border likely play roles in the overall morphology of the ruffled border. This sort of interaction has been described for certain ATP synthases, specifically those from the mitochondria of *Euglena gracilis* [137]. ATP synthases in general are close relatives of V-ATPases [46].

## 8. Direct Response of Osteoclasts to Mechanical Stimulation

Mechanical strain is well known to affect the bone structure and has been extensively studied with respect to its effects on osteocytes and osteoblasts [138]. Osteocytes respond to mechanical stimulation by changes in the production of sclerostin by osteocytes, which then regulates WNT/β-catenin signaling in osteoblasts [21] as well as other signaling pathways [21]. Two routes by which osteocytes sense mechanical signaling include β1 integrins [139,140] and transient receptor potential cation channel subfamily V member 4 (TRPV4) [141], but there remain significant knowledge gaps in this area. Osteoblasts directly respond to mechanical stimulation by changes in numerous signaling networks [142], although they are less responsive than osteocytes. Osteoclasts have not been studied as extensively, but in general, it has been shown that mechanical stimulation reduces osteoclast formation and bone resorption when other factors are constant, consistent with the theme that mechanical stimulation is bone anabolic [143,144,145]. With the recent studies that suggest that osteoclasts are full partners, with osteoblasts and osteocytes, in signaling involved in the maintenance of the bone [18], new studies examining how mechanical signaling affects intercellular signals generated by osteoclasts are warranted.

## 9. Summary

Osteoclasts display profound responses to their substrate. Osteoclasts in the bone, dentine, and certain other mineralized surfaces undergo dramatic reorganization of their cytoskeleton and membrane trafficking machinery, leading to the formation of a resorption apparatus, which includes a ruffled border and actin ring. Integrins are known to be involved in detecting signals from the substrate that promote the activation of osteoclasts. Additional receptors in osteoclasts are likely involved in regulating activation, although their roles are less well understood. Deeper understanding of the signals that osteoclasts receive from the bone has the potential to lead to new therapeutics for modulating bone remodeling and the construction of new materials for replacement of the bone.

## Abbreviations

Arp2/3	Actin-related 2/3
V-ATPases	Vacuolar H+-ATPases
CLC-7	Chloride voltage-gated channel 7
OSTM1	Osteopetrosis-associated transmembrane protein 1
RGD	Arginine-glycine-aspartic acid
c-src	Cellular-proto-oncogene tyrosine-protein kinase Src
PILP	Polymer-induced liquid precursor
LRP1	LDL-receptor-related protein 1
TRPV4	Transient receptor potential cation channel subfamily V member 4
EV	Extracellular vesicle
RANK	Receptor activator of nuclear factor kappa B
RANKL	Receptor activator of nuclear factor kappa B-ligand
MT1-MMP	Membrane type 1-matrix metalloproteinase
CAP	Capping protein
